# Nurses’ Knowledge and Practice of Appropriate Techniques of Body Mechanics and Non-specific Back Pain

**DOI:** 10.7759/cureus.56478

**Published:** 2024-03-19

**Authors:** Haya Douhal, Samiha Jarrah, Rami Masa'deh, Raed Shudifat

**Affiliations:** 1 ICU/CCU Nurse, Royal Hospital, Amman, JOR; 2 Faculty of Nursing, Applied Science Private University, Amman, JOR; 3 Adult Health Nursing Department, Mutah University, Karak, JOR

**Keywords:** non-specific back pain, body mechanics, nurses, practice, knowledge

## Abstract

Background

Nursing is a compassionate profession that carries occupational hazards, including work-related injuries. Back pain is a common concern due to the physically demanding tasks performed by nurses. Utilizing proper techniques of body mechanics is vital to prevent work-related back pain and enhance overall well-being.

Aim

To assess the knowledge and practice of the nurses working in Jordanian hospitals about the appropriate techniques of body mechanics and their relation to non-specific back pain.

Methods

A cross-sectional design with a convenience sample of 280 participants was randomly selected from hospitals. The tool included the use of a paper questionnaire or scanning the barcode at nursing stations; a reliable, adopted tool was used in this study.

Results

Out of 280 participants, six (2.1%) had poor knowledge, 96 (34.3%) had average knowledge, and 178 (63.6%) had good knowledge, with a mean score of 3.72±0.58. For practice, seven (2.5%) had poor, 225 (80.4%) had average, and 48 (17.1%) had good practice, with a mean score of 3.30±0.49. Both knowledge and practice scores were negatively correlated with non-specific back pain (rpb = -.393 and rpb = -.306, p < .001), respectively. Furthermore, sociodemographic characteristics did not significantly affect body mechanics knowledge and practice scores (p > 0.05) for all variables.

Conclusion

Nurses working in Jordanian hospitals had good knowledge of body mechanics but average practice. Higher knowledge and practice levels were correlated with less experience of non-specific back pain. Additionally, there was no significant difference in sociodemographic data between nurses with knowledge and practice scores.

## Introduction

One of the most prevalent occupational health issues among nurses is back pain [1-3]. Registered nurses who manually lift and move patients and experience musculoskeletal illnesses ranked fifth [4]. Due to the demanding and heavier loads of their occupational activities, healthcare employees, particularly nurses, are more likely to experience low back pain and other musculoskeletal problems [5-7]. Like Jafar and AN mentioned in their study, a musculoskeletal injury may cause a nurse's career to suffer; many nurses who would want to provide direct patient care have been forced to leave nursing or the style of nursing they would prefer due to injury [8].

Body mechanics can be referred to as how humans move throughout the course of a typical day [8]. It covers the way our bodies are held while we stand, sit, lift, carry, bend, and sleep. Poor body mechanics cause a lot of problems, such as back pain. Having a good understanding of body mechanics and how to use it effectively in daily practice is a necessity for nurses [9]. Different authors have defined body mechanics as the process of effectively using one's body to do actions; learning the proper motion, such as bending, lifting, stretching, sitting, standing, or reclining while carrying out duties, is crucial [9-11]. Examples of poor body mechanics that can cause low back pain include standing for a long time, moving or lifting heavy objects, and bending or twisting incorrectly [11].

This study aims to assess the knowledge and practice of nurses working in Jordanian hospitals regarding appropriate techniques of body mechanics and their relation to non-specific back pain. The study is expected to address the following: 1. the level of knowledge among registered nurses about proper body mechanics in Jordanian hospitals; 2. the level of practice among registered nurses about proper body mechanics in Jordanian hospitals; 3. the relationship between the level of knowledge and practice among registered nurses about proper body mechanics and non-specific back pain in Jordanian hospitals; and 4. the difference in the level of knowledge and practice about proper body mechanics based on sociodemographic factors (department, age, gender, professional education, and job experience) in Jordanian hospitals.

## Materials and methods

A cross-sectional descriptive study assessed knowledge and practice of appropriate body mechanics and non-specific back pain among registered nurses in Jordan. Three hospitals were randomly selected, excluding Royal Medical Services and teaching hospitals due to the long review process. Hospitals were chosen from random number tables: Royal Private Hospital (private sector), Al-Basheer Public Hospital, and Prince Hamza Hospital representing intensive care units, medical and surgical floors, and orthopedic floors with high bedridden patient prevalence.

Sample

Registered nurses with a bachelor's degree or higher who provide direct patient care were included in the study. Matrons/nursing managers, and nurses with specific back pain were excluded from the study.

The sample size was calculated using G Power software, taking into consideration the main statistical tests used in the study (i.e., t-tests and analysis of variance (ANOVA)), the need for a power of 0.90, a medium effect size of 0.25, and a level of significance of 0.05. Therefore, 255 participants were sufficient to determine statistically significant results. This study included 280 participants, accounting for a 10% chance of attrition.

Data collection

Following IRB approval from Applied Science Private University, designated hospitals were visited. The head nurses of the intensive care units, medical and surgical floors, and orthopedic floors were contacted and explained the proposal and study purpose, obtaining their approval. A poster, including the study title, purpose, researcher name, contact information, and a barcode, was displayed in their departments for online questionnaire access. Paper questionnaires were also available in a box next to the poster at the nursing station. Respondents were informed to take their time answering the survey.

Measurement of variables

To assess the knowledge and practices of appropriate body mechanics and non-specific back pain among nurses, a well-constructed questionnaire was adopted to collect the data [12]. The adopted questionnaire was based on the Thurston scale ‘yes’ and ‘no', which was converted to a five-point Likert scale. Cronbach’s alpha was checked, resulting in .80 for the knowledge questions and .70 for the practice questions, indicating reliability [12].

Participants responded on a five-point Likert scale ranging from 1 to 5, where 1 is strongly disagree, 2 is disagree, 3 is neutral, 4 is agree, and 5 is strongly agree. The mean scores of knowledge and practice are categorized as follows: (1-2.33) poor knowledge/practice level, (2.34-3.67) average knowledge/practice level, and (3.68-5) good knowledge/practice level, based on a cutoff point for the five-point Likert scale that classifies on three-type scales [13].

Data analysis

Questions one and two will be analyzed using descriptive statistics (percentages, frequencies, mean, and standard deviation). Research question three will utilize correlation analysis, as knowledge and practices of body mechanics are measured on a scale level, while non-specific back pain is measured in binary form using point biserial correlation. Research question four will employ inferential statistics, specifically T-tests and ANOVA.

## Results

A total of 280 registered nurses were enrolled in the study; 137 were males (48.9%) and 143 were females (51.1%). The majority of the sample had a bachelor’s degree in nursing, with 244 (87.1%) compared to 36 (12.9%) having completed higher studies. Approximately one-quarter of nurses were in age groups 21-25, 26-30, and 31-35 years, respectively, reflecting that over 75.0% of them were less than 35 years old. Furthermore, the majority had working experience of less than five years (127, 45.4%), while only 14.3% had experience above 15 years. About half of the nurses worked in the medical and surgical departments, and 215 (76.8%) experienced non-specific back pain. Please refer to Table 1 for sample characteristics.

**Table 1:** Frequencies and percentages of sample characteristics (N=280)

Variables	Category	Frequency	Percentage (%)
Gender	Male	137	48.9
	Female	143	51.1
Education level	Bachelor	244	87.1
	Higher studies	36	12.9
Age in years	21-25	77	27.5
	26-30	70	25.0
	31-35	72	25.7
	36-40	37	13.2
	Above 40	24	8.6
Work experiences in years	0-5	127	45.4
	6-10	59	21.1
	11-15	54	19.3
	above15	40	14.2
Department	ICU	113	40.4
	Medical/surgical	138	49.3
	General orthopedic	29	10.3
Experience non-specific back pain	No	65	23.2
	Yes	215	76.8

The researcher utilized a 15-item knowledge scale among nurses regarding proper body mechanics. Options were grouped (agree/strongly agree; disagree/strongly disagree). Frequencies, percentages, mean, and standard deviation were computed for three options (Table 2). Results showed that 70.4% of nurses with good knowledge of body mechanics had advantages in reducing back pain, maintaining body function (75.0%), reducing strain/spasm (75.4%), and maintaining body balance (74.3%). Additionally, 74.6% had good knowledge regarding using bed sheets instead of hands when lifting patients, and 70.4% agreed that proper shoes influence back pain. Regarding musculoskeletal predisposing factors, participants agreed that poor posture leads to back pain (74.3%), continuous muscle tension (81.1%), heavy work activities (74.6%), and improper body mechanics cause musculoskeletal pain or spinal injuries (71.1%). Only 40.7% agreed on understanding the body mechanics concept. The total knowledge mean score was (3.72±0.58), indicating good knowledge. Six participants (2.1%) had poor knowledge, 96 (34.3%) had average knowledge, and 178 (63.6%) had good knowledge among 280 participants.

**Table 2:** The level of knowledge among registered nurses about proper body mechanics (N=280)

Item description	Agree n(%)	Neutral n(%)	Disagree n(%)	Mean ± SD
1. Use of body mechanics can reduce back pain	197 (70.4)	48 (17.1)	35 (12.5)	3.68±1.06
2. Lifting heavier patients using bed sheets is better than using hands	209 (74.6)	37 (13.2)	34 (12.1)	3.83±1.08
3. I have back pain if I don’t maintain good posture while doing procedures	208 (74.3)	50 (17.9)	22 (7.9)	3.91±0.99
4. Body mechanics practices maintain proper body function	210 (75.0)	47 (16.8)	23 (8.2)	3.83±0.90
5. Body mechanics practices reduce strain/spasm	211 (75.4)	45 (16.1)	24 (8.6)	3.81±0.90
6. Body mechanics maintain the balance	208 (74.3)	554 (19.3)	18(6.4)	3.80±0.80
7. Use of continuous muscle tension causes injuries and musculoskeletal pain	227 (81.1)	33 (11.8)	20 (7.1)	4.00±0.91
8. Object must be close to the center of gravity (close to your body)	161 (57.5)	74 (26.4)	45 (16.1)	3.52±0.98
9. The principle of body mechanics is that the act of “attractive” may produce less injury	159 (56.8)	89 (31.8)	32 (11.4)	3.53±0.90
10. Injuries can be avoided through appropriate body mechanics	193 (68.9)	44 (15.7)	43 (15.4)	3.66±0.96
11. I know the purpose of using body mechanics	191 (68.2)	63 (22.5)	26 (9.3)	3.71±0.85
12. Heavy work activities like bending, twisting, and frequent heavy lifting contribute to low back pain	209 (74.6)	31 (11.1)	40 (14.3)	3.88±1.01
13. Improper use of body mechanics techniques causes spinal injuries	199 (71.1)	49 (17.5)	32 (11.4)	3.73±0.97
14. Attire (shoes) play an important role in influencing back pain	197 (70.4)	52 (18.6)	31 (11.1)	3.76±0.96
15. I know what body mechanics is all about	114 (40.7)	112 (40.0)	54 (19.3)	3.22±0.94
Total knowledge mean score				3.72±0.58

The researcher utilized a six-item nurses' practice scale for proper body mechanics. Responses were categorized as agree and strongly agree or disagree and strongly disagree, with frequencies, percentages, mean, and standard deviation computed for each (Table 3). Approximately 81.0% of nurses requested assistance when handling obese patients. However, only 24.7% and 28.2% demonstrated the proper technique in using lower body extremities for lifting objects or moving patients in bed. Moreover, about 64.2% applied body mechanics principles when transferring patients from bed to chair, while 62.1% wore appropriate footwear during work hours. Notably, 31.5% consistently practiced body mechanics while working. The overall mean practice score was 3.30±0.49, indicating an average level of adherence to proper body mechanics among nurses. Specifically, seven out of 280 participants (2.5%) exhibited poor practice, 225 participants (80.4%) showed average practice, and 48 participants (17.1%) demonstrated good practice.

**Table 3:** The level of practice among registered nurses about proper body mechanics (N=280) * Reversed items

Item description	Agree n(%)	Neutral n(%)	Disagree n(%)	Mean ± SD
1. I ask for help from a colleague if I have to lift an obese patient	227 (81.0)	34 (12.1)	19 (6.8)	4.11±0.95
2*. I straighten my knees and bend back when lifting an object from the floor	146 (52.1)	65 (23.2)	69 (24.7)	2.74±1.01
3*. I close my leg when moving the patient in bed	130 (46.4)	71 (25.4)	79 (28.2)	2.76±1.03
4. I use the principle of body mechanics during the procedure for removing the patient from bed to chair	180 (64.2)	58 (20.7)	42 (15.1)	3.61±0.97
5. I wear proper attire (shoes) during working time	174 (62.1)	54 (19.3)	52 (18.6)	3.59±1.01
6. I practice body mechanics all the time during working time	88 (31.5)	99 (35.3)	93 (33.2)	2.98±1.02
Total practice mean score				3.30±0.49

The results presented in Table 4 revealed that knowledge and practice scores were negatively significantly correlated with non-specific back pain (rpb = -0.393 and rpb = -0.306, p < 0.001), respectively, indicating that higher knowledge and practice scores were associated with a lower incidence of non-specific back pain.

**Table 4:** The relationship between the level of knowledge and practices among registered nurses about proper body mechanics and non-specific back pain (N=280) * α ≤ 0.05

Proper body mechanics		Non-specific back pain
Knowledge level	Point biserial correlation	-.393
	Sig. (2-tailed)	< .001>
	N	280
Practice level	Point biserial correlation	-.306
	Sig. (2-tailed)	< .001>
	N	280

The results presented in Table 5 demonstrated that the nurses' knowledge scores regarding proper body mechanics were similar and not significantly different to reach statistical significance. Therefore, the proper body mechanics knowledge scores did not vary significantly based on nurses' socio-demographic characteristics (p > 0.05) across all variables.

**Table 5:** The differences in knowledge about proper body mechanics based on socio-demographics (gender, education level, experience, age, and department) (N=280)

Variables	Category	Mean	SD	Test value	p-value
Gender	Male	3.66	0.61	1.735	0.084
	Female	3.78	0.55		
Education level	Bachelor	3.72	0.57	0.120	0.904
	Higher degrees	3.74	0.66		
Work experience in years	0-5	3.81	0.57	2.299	0.078
	6-10	3.62	0.57		
	11-15	3.60	0.59		
	Above 15	3.78	0.60		
	Age in years	21-25	3.83	0.62	0.987	0.415
26-30		3.67	0.53		
31-35		3.68	0.56		
36-40		3.68	0.58		
Above 40		3.73	0.66		
Department	ICU	3.69	0.63	1.315	0.270
	Medical/surgical	3.78	0.52		
	General	3.61	0.65		
	Orthopedic				

Also, the practice scores of registered nurses regarding proper body mechanics were similar, converged, and not significantly different, indicating that nurses' demographic characteristics did not affect proper body mechanics practice scores (p > 0.05) for all variables (Table 6).

**Table 6:** The differences in the practice of proper body mechanics based on socio-demographics (gender, education level, experience, age, and department) (N=280)

Variables	Category	Mean	SD	Test value	p-value
Gender	Male	3.25	0.48	1.506	0.133
	Female	3.34	0.50		
Education level	Bachelor	3.31	0.48	0.983	0.326
	Higher degrees	3.22	0.57		
Work experience in years	0-5	3.32	0.50	0.315	0.815
	6-10	3.28	0.52		
	11-15	3.25	0.46		
	Above 15	3.30	0.49		
Age in years	21-25	3.27	0.54	1.311	0.266
	26-30	3.31	0.43		
	31-35	3.33	0.49		
	36-40	3.16	0.52		
	Above 40	3.44	0.48		
Department	ICU	3.25	0.45	0.994	0.371
	Medical/surgical	3.34	0.51		
	General orthopedic	3.28	0.56		

## Discussion

The study aimed to assess nurses' knowledge and practice regarding proper techniques of body mechanics and their relation to non-specific back pain in Jordanian hospitals. Body mechanics pertains to how we use our bodies in daily activities such as bending, standing, sitting, and lifting objects or individuals [12,14]. In this study, out of 280 participants, 137 were males (48.9%) and 143 were females (51.1%). The majority (87.1%) held a bachelor's degree in nursing, while 12.9% had higher education qualifications. Additionally, 75.0% of the sample were below 35 years old, with 53% aged between 21 and 30 years and 36% below 30 years, with an average age of 35.7. Also, in the present study, about half of registered nurses work in medical/surgical departments, and 215 (76.8%) of the nurses experienced non-specific back pain. Rawat et al. revealed that 21% of study participants experienced back pain, out of which 13% stated that the back pain was caused by heavy lifting [15].

In this study, the total knowledge mean score was found to be 3.72±0.58, indicating the nurses’ level of knowledge regarding proper body mechanics, which is considered good according to the cut-point scale [13]. Out of 280 participants, 178 (63.6%) had good knowledge, 96 (34.3%) had average knowledge, and six (2.1%) had poor knowledge. This finding is consistent with Akhtar et al.'s study conducted at the Punjab Institute of Cardiology in Lahore to assess the knowledge and practice of appropriate techniques of body mechanics among 216 nurse participants, which found that 140 (65%) have good knowledge and 45 (20%) have average knowledge [12]. This is similar to a study that occurred in a college of nursing in India about the knowledge of nurses about body mechanics, which showed that 41.7% of nurses had good knowledge of the appropriate techniques of body mechanics [4]. In the present study, out of 280 participants, seven (2.5%) had poor practice, 225 (80.4%) had average practice, and 48 (17.1%) had good practice. The total practice mean score was 3.30±0.49, reflecting nurses’ practice level regarding proper body mechanics, which was an average level according to the cut-point scale [13]. Similar results were found in a correlational study conducted at a nursing college in Mangalore, India, where 88% of the participants had average practices towards body mechanics [16]. And according to Kumar & Damanpreet, 52.9% had average practices and 24.1% had poor practices [14]. In contrast, a study involving 216 participants at the Punjab Institute of Cardiology, Lahore, to assess knowledge and practice of the appropriate techniques of body mechanics among nurses revealed that 60% had good practice and 30% had average practice [12].

The findings indicate that nurses in Jordanian hospitals with higher knowledge and practice levels of appropriate techniques of body mechanics experienced less non-specific back pain. This aligns with a correlational study showing a positive correlation between a lack of knowledge and practice regarding body mechanics and low back pain [17]. Similarly, another study [18] found a statistically positive correlation between barriers to performing body mechanics, such as inadequate lifting equipment, and the severity of back pain. Additionally, Rochman et al. mentioned that knowledge of body biomechanics had the strongest correlation with low back pain incidence among relevant characteristics [19]. Furthermore, other studies [20,21] revealed that nurses performing interventions involving bending forward, lifting and transferring patients, and working in standing postures for extended periods scored higher on average for back pain.

The study results showed nurses' knowledge scores on proper body mechanics were similar across demographics, with no significant difference in practice scores based on socio-demographic characteristics (p > 0.05). This aligns with other studies indicating no association between knowledge/practice of body mechanics and selected demographics (gender, education, clinical experience, age) [11,15,22,23]. In contrast, there was a significant correlation between using body mechanics concepts and nurses' years of experience [18,24].

Limitations of the study 

The convenient sampling and cross-sectional design may affect the generalizability of the findings. Also, different settings are needed to be able to generalize the findings, as only three hospitals were included. There was a short time for data collection as obtaining ethical approvals was difficult.

## Conclusions

The study revealed that the 280 nurses who are working in Al-Basheer Public Hospital, Prince Hamza Public Hospital, and Royal Private Hospital showed a good knowledge level of proper body mechanics, while their practice score was at an average level. More than three-quarters of participants experience non-specific back pain that could mainly be related to work or improper body mechanics. Also, there is a significant negative correlation between knowledge and practice of body mechanics and experience of non-specific back pain; higher knowledge and practice of body mechanics are correlated with lower experience of non-specific back pain, and the results of the study demonstrated there is no significant difference between knowledge score and practice score with sociodemographic characteristics (department, age, gender, education level, and job experience), meaning the demographics of the nurses had no apparent impact on their knowledge and practice scores for proper body mechanics.
